# Prevalence of avian infectious bronchitis virus in broiler chicken farms in south of Iraq, 2014 – 2015

**Published:** 2016-12-15

**Authors:** Waleed Seger, Arash Ghalyanchi Langeroudi, Vahid Karimi, Omid Madadgar, Mehdi Vasfi Marandi, Masoud Hashemzadeh

**Affiliations:** 1 Department of Pathology and Poultry Diseases, Faculty of Veterinary Medicine, University of Basra, Basra, Iraq; 2 Department of Microbiology and Immunology, Faculty of Veterinary Medicine, University of Tehran, Tehran, Iran; 3 Department of Poultry Diseases, Faculty of Veterinary Medicine, University of Tehran, Tehran, Iran; 4 Department of Research and Production of Poultry Viral Vaccine, Razi Vaccine and Serum Research Institute, Karaj, Iran

**Keywords:** Avian infectious bronchitis, Broiler, Iraq, Real-time RT-PCR

## Abstract

Avian infectious bronchitis (IB), caused by a gammacoronavirus, is an OIE-listed (List B) disease and characterized by respiratory and renal involvements, causing high mortality, and economic loss in both layers and broilers. In comparison with other diagnostic methods, real-time polymerase chain reaction (RT-PCR) and conventional RT-PCR are potent, more sensitive and faster techniques for infectious bronchitis virus (IBV) detection. This research was conducted to detect IBV using specific primers of IB in three governorates (Basra, Thi-Qar and Muthana) in the south of Iraq. Tracheal specimens were collected from 46 IB suspected commercial broiler flocks. XCE2+ and XCE2- Primers, which amplify all IBV serotypes, were used. Primers MCE1+, BCE1+ and DCE1+ were used to amplify the specific nucleotide sequences of Massachusetts, 793/B and D274 genotypes, respectively. The results of real-time RT-PCR of this study showed that 34 (74.00%) out of 46 infected flocks were positive to IBV. The results of nested PCR showed that 50.00% and 5.89% of positive samples were belonged to genotypes 793/B and Massachusetts, respectively, and the remaining positive (44.11%) were unknown. The results indicate presence of Massachusetts and 793/B IBV strains in commercial broilers in southern Iraq.

## Introduction

Infectious bronchitis (IB) is a highly contagious viral disease of the upper respiratory and urogenital tract of chickens, which is caused by infectious bronchitis virus (IBV). The disease is prevalent in all countries with an intensive poultry industry, affecting the performance of both broilers and layers, thereby causing the considerable economic loss in poultry industry worldwide.^1^ The virus is the coronavirus of the domestic fowl that is mainly observed in chicken. It possesses a positive sense single–stranded RNA genome that ranges from 27 to 31 Kb.^2^ The number of IBV serotypes that exist throughout the world is unknown. More than 50 different serotypes have been listed and new IBV variants continue to emerge.^3^ It is now well documented that a considerable number of different serotypes with antigenic and pathogenic differences exist in poultry industry of different parts of the world.^4^

The D274 type was the most common type of IBV in several western European countries in the early and mid-1980s.^5^ IBV strains of the 4/91 type, which are also known as 793B, were first reported and characterized in Britain, 1991,^6^ and have been the dominant genotype in Europe.^7^ The Serological survey has revealed a high incidence of IBV infection of the 793/B type in layer and broiler chickens worldwide.^8^ Infectious bronchitis still causes serious problems in the Iraqi poultry industry due to the inability of the vaccines to protect the different genotypes. Due to the limited network of poultry diagnostic laboratories in Iraq, differential diagnosis is only made according to clinical signs and gross lesions.

The characterization of IBV has raised additional problems in terms of both epidemiology and control. Although IBV in the poultry farms in Iraq (with H120 and 4/91 strains) is presently controlled by both inactivated and live attenuated vaccines, the outbreaks of IB have still been observed on broiler farms.^9^^,^^10^ In Iraq, the first report of identification and genotyping of IBV isolates has been from Kurdistan-Iraq, which indicated the circulation of 793/B ( with the prevalence rate of 25 %) along with a new IBV variant (Sul/01/09) in vaccinated (Ma5 , or 4/91) broiler farms.^10^ So far, there has been no report on the prevalence rate of IBV genotypes in the south of Iraq.

The aim of the present study was to detect of three IBV genotypes including (Massachusetts; Mass), 793/B and D274 in the south of Iraq.

## Materials and Methods

**Sampling.** Tracheal tissue samples were collected from 46 IB suspected broiler farms, located in Basra, Thi-Qar and Muthana governorates. All chicks were 20-35 day-old and vaccinated with H120 and 4/91. Five tracheal tissue samples per flock were selected for RNA extraction.

**RNA extraction and cDNA synthesis. **Total RNA was extracted from the tracheas using CinnaPure RNA (Sinaclon Co., Tehran, Iran) based on the kit instructions. The extracted RNA was used in reverse transcription (RT) reaction to generate cDNA through cDNA synthesis kit (Thermo scientific, Waltham, USA). The cDNA was stored at –20 ˚C until use.

**Real-time PCR for IBV detection. **Real-time PCR was carried out to detect IBV. TaqMan^®^ probe and primers (Bioneer Corporation, Daejeon, Korea) were used in this study according to Callison et al. method.^11^ Forward primer 5’ GCTTTTGAGCCTAGCGTT3’, reverse primer 5’ GCCATGTTGTCACTGTCTATTG 3’, and TaqMan^®^ dual-labeled probe FAM-CACCACCAGAACCTGTCACCTC-BHQ1, were used to amplify and detect a 143-bp fragment of the 5’-untranslated region (5’UTR). The mixture for each tube was consisted of 13 μL TaqMan Master Mix, 0.2 μL of 2 pM IBV probe, 1 μL of 10 pM forward primer, 1 μL of 10 pM reverse primer, 2.8 μL distilled water, to reach a total volume of 18 μL. Then, 2 μL cDNA was added. A negative control containing nuclease-free water instead of cDNA were included in each run. The thermal profile for the PCR was 95 ˚C for 10 min, followed by 40 cycles of 94 ˚C for 15 sec, 50 ˚C for 30 sec and 72 ˚C for 30 sec. PCR amplification was performed using Rotor Q (Qiagen Co., Hilden, Germany) system.

**Nested PCR for IBV genotyping. **For determination of genotypes, a type specific nested PCR was conducted.^12^ Oligonucleotide primers included MCE1+, DCE1+, and BCE1+ which specifically amplified a hyper-variable region of the S1 gene of Mass, D274 and 793/B serotypes, respectively. XCE3- primer was a common primer for all IBV genotypes detection. Primer sequences and their expected band size are shown inTable 1. The first round amplification was performed in a final volume of 20 µL (2 μL distilled water, 13 μL 2X PCR master mix (Sinaclon Co.), 1 μL forward primer (XCE2+) primers, 1 μL reverse primer (XCE2-) and 3 μL cDNA. Amplification was performed with a thermal profile of (94 ˚C for 5 min, 94 ˚C for 45 sec, 58 ˚C for 45 sec, 72 ˚C for 90 sec, and 72 ˚C for 5 min) for 40 cycles. Two microliter of the first round PCR product was diluted with 198 μL of distilled water, whereas in the second round nested PCR we used (2 μL distilled water, 13 μL 2X PCR master mix (Sinaclon Co.), 1 μL forward Primer (XCE3-), 1 μL reverse primer (DCE1+) and or MCE1+, BCE1 + and 3 μL RT-PCR Product) amplification was 94 ˚C for 2 min, 94 ˚C for 15 sec, 48 ˚C for 30 sec, 68 ˚C for 30 sec, and 68 ˚C for 10 min) for 40 cycles. The polymerase chain reaction (PCR) product was analyzed by electrophoresis on 1% agarose gel.

## Results

**Fig. 1:**
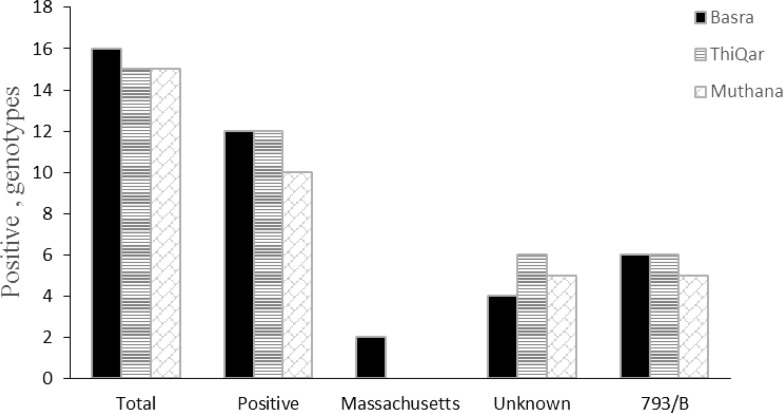
Distribution of positive IBV samples including 793/B, Massachusetts and unknown strains in the broiler farms of three governorates in the south of Iraq

**Fig. 2:**
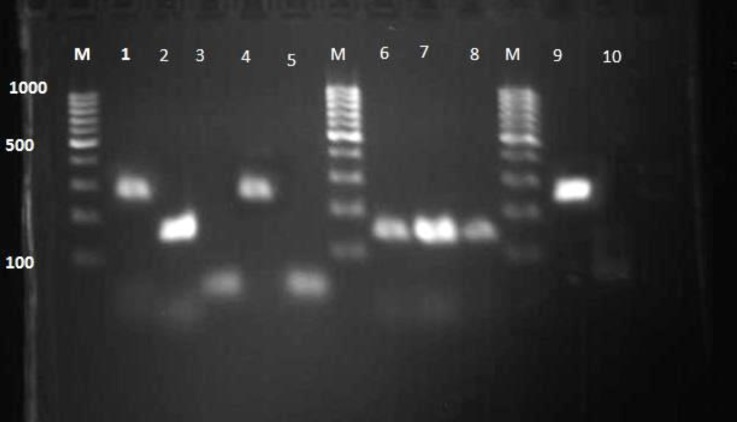
Electrophoresis (2% agarose gel) for infectious bronchitis virus (IBV). **Lane M:** 100 bp – 1000 kbp DNA ladder marker; **Lane 1:** Massachusetts (positive control; band at 295 bp); **Lane 2:** Positive control (793/B; band at 154 bp); **Lanes 3: **Negative control; **Lane 6, 7, 8: **793/B (positive samples); **Lanes 4 and 9:** Massachusetts (positive samples); **Lanes 5 and 10:** Negative samples

In the current research, 46 broiler flocks were selected for tracheal tissue sample collection. The results of real-time RT-PCR of this study showed that 34 (74.00 %) out of 46 infected flocks were positive to IBV (Fig. 1). 35.00% of positive samples were from Basra, 35.00% of them were from Thi-Qar and 30.00% from Muthana governorates. Amplification of an expected DNA band of 295 bp, and 154bp from positive control, as well as, positive samples indicated that the nested PCR reaction has been performed correctly (Fig. 2). The results of nested PCR revealed that (50.00%), (5.89%), and (44.11%) of these flocks have 793/B, Mass, and unknown strains, subsequently (as shown in Fig. 1). 793/B had the equal prevalence rate in each governorate, but Mass serotype was just detected in Basra governorate. The present study indicated a relatively high prevalence of 793/B IBV genotype in the south of Iraq. D274 genotype was not detected.

**Table 1.** Sequences of oligonucleotides used as primers in the nested RT-PCR.^12^

**Primer**	**Sequence (5' -'3)**	**Amplification fragment (bp)**
**XCE2+**	5'-CACTGGTAATTTTTCAGATGG-3’	466
**XCE2-**	5'- CCTCTATAAACACCCTTGCA-3’	466
**DCE1+**	5'- TTCCAATTATATCAAACCAGC-3'	217
**MCE1+**	5'- AATACTACTTTTACGTTACAC-3'	295
**BCE1+**	5'-AGTAGTTTTGTGTATAAACCA-3'	154

## Discussion

Infectious bronchitis (IB) has a significant economic impact on the poultry industry causing the loss of millions of dollars worldwide each year due to the condemnation of infected chickens.^13^ Since it was first described in the early nineties in the United Kingdom, 4/91 type IBVs was identified in many other countries and became one of the most predominant genotype in Europe.^14^^,^^15^ Recombination with field isolates and reversion to virulence has been reported for the IBV vaccines.^16^^,^^17^ Therefore, it is important to verify the relationship between field isolates and the vaccine strain.^18^ Despite the availability of an attenuated vaccine against 793/B serotype viruses, recent studies suggest that viruses belonging to the 793/B sero-group are still prevalent in European flocks, and their continual presence is still an important concern for the poultry industries in many different countries.^19^ It is now recognized that IBV comprises more than one serotype. The original serotype Mass has now been spread worldwide and is included in most of the commercially available vaccines.^20^

The results of the present study showed that 34 (74.00%) out of 46 sampled flocks were positive to IBV by real-time PCR and that 35.00%, 35.00% and 30.00% of the sampled flock in Basra, Thi-Qar and Muthana, respectively, governorates were positive to IBV. In addition, the results showed the distribution of 793/B and Mass genotypes with the prevalence rate of 50.00% and 5.89%, respectively, yet no D274-type virus was detected in the south of Iraq. The presence of other IBV types is probable, however, they were not detectable as applied primers were specifically amplified three types of IBV. Unknown positive IBV strains must be sequenced to be characterized.

In Israel, Mass was the only type detected for many years until the 793B type of IBV (Israel/793B/variant 1/96) was identified in 1996.^21^ Detection of 793/B serotype has been reported from Saudi Arabia, Japan, Denmark, Poland, France, Italy, and Argentine.^22^ Also, the 793B type was reported in Iran; the co-existence of Mass and 793/B type’s viruses at the same time in 33 flocks in Iran revealed that Mass type vaccine viruses have not conferred a proper immunity against 793/B type viruses.^23^ There was a study in the middle of Iraq, showed that 92.10% of samples collected from suspected flocks were infected with IBV, 20.00% of samples were infected with IB alone and 45.71% of samples with IB combined with both *Mycoplasma galisepticum* and avian influenza virus subtype H9 and 25.71% of samples were positive to both IBV and AIV(H9).^9^ Mahmood *et al.* isolated infectious bronchitis virus from trachea and kidney tissues of eight broiler farms in Kurdistan region of North Iraq from 2008 to 2010. Their results showed that two farms were just infected with 793/B type, one with an unknown strain and five with Sul/ 01/09.^10^ Jahantigh *et al.* conducted a work to identify the infectious bronchitis virus with group-specific primers in Zabol, south-east of Iran. Their results showed that 36.36% of the sampled flocks were positive to IBV by RT-PCR.^24^ Also, in Jordan, there are studies that have detected the presence of IBV, like Ark, DE-072, and Mass-like serotypes.^25^ Another study of Jordan showed that sampled flocks have Mass, 4/91, and D274 isolates, respectively.^26^ However, D274 genotype was not detected in the present study.

Our results showed the low prevalence (5.89%) of Mass type in Basra that was in consistent with Mahmood *et al.* results who found no Mass-type IBV in the North of Iraq.^10^ In our research, 50.00% of farms in each governorate were infected with type 4/91, which was two times higher than its prevalence in Kurdistan region of Iraq. According to 4/91 based vaccination strategies applied in Iraq, 4/91-type IBVs could be vaccine-derived, however, this high prevalence rate also may reflect the weak biosecurity systems and uncontrolled entering of broiler chicks from other Iraqi governorates or intense trading with neighboring countries (Turkey, Iran, Jordan, and Saudi Arabia).

The large number of IBV variants exist worldwide, some being unique to a particular area and others having a more general distribution. The reason why some strains spread readily over major parts of the world whereas others remain local is unknown. Some IBV variants, such as 793/B or QX that have been spread over Asia, Europe, and Africa in a short period, have not been reported in the USA or Australia. It seems likely that geographical isolation and control measures employed in countries may play a significant role in preventing entry of IBV variants. The recent discoveries of IBV and IBV-like strains in other species of birds other than the chicken,^27^ like geese, ducks, and pigeons, might also play roles in the spread of IBV strains over the world. Specific IBV strains that were able to infect another bird species, especially if it is a migratory bird, would spread more easily over long distances than a strain that could not replicate in that bird species.

In conclusion, the current study clearly demonstrated that there was the presence of 793/B and Mass IBV genotypes (Vaccine or field strain) in commercial chicken flocks in south of Iraq. By utilizing diagnostic techniques such as those were used in this study, it is possible to conduct a detailed epidemiological investigation of this disease. Despite the use of H120 and 4/91 vaccines in Iraq, it is common to find IB problems in vaccinated chickens. In the present study, the sequencing of PCR products was not performed, therefore, the origin of these types is not clear at present. Future work should include the sequencing of IBV strains in the region in order to determine the type of unknown strains and to choose the suitable vaccination program.
